# The role of E3 ubiquitin ligases in selective types of macroautophagy

**DOI:** 10.1038/s44319-026-00887-1

**Published:** 2026-07-23

**Authors:** Monja Müller, Konstanze F Winklhofer, Fulvio Reggiori

**Affiliations:** 1 Department of Biomedicine, Aarhus University, Høegh-Guldbergsgade 10, 8000 Aarhus C, Denmark; 2 Department Molecular Cell Biology, Institute of Biochemistry and Pathobiochemistry, Ruhr University Bochum, 44801 Bochum, Germany; 3 Cluster of Excellence RESOLV, Ruhr University Bochum, 44801 Bochum, Germany

**Keywords:** Autophagy & Cell Death, Organelles, Post-translational Modifications & Proteolysis

## Abstract

In contrast to the ubiquitin (Ub)-proteasome-system, which only degrades individual proteins, macroautophagy can eliminate protein complexes or aggregates, organelles and even pathogens. Terms such as mitophagy, aggrephagy, lysophagy and xenophagy have been coined based on the targeted substrate. In Ub-dependent selective macroautophagy, cargo selectivity is specified by E3 Ub ligases that append Ub chains that in turn are recognized by selective autophagy receptors (SARs), driving sequestration into autophagosomes. While several Ub-dependent SARs have been identified and characterized, the E3 Ub ligases that ultimately decide target fate remain poorly studied. In this review, we summarize what is known about the E3 Ub ligases involved in selective macroautophagy, with a particular emphasis on the degradation of mitochondria, protein aggregates, lysosomes and pathogens. A better characterization of these enzymes could improve therapeutic strategies for targeted degradation in acute and chronic diseases.

## Abstract (web summary)

A subset of selective autophagy receptors recognizes ubiquitin modifications on their targets. This review provides a comprehensive overview on E3 ubiquitin ligases involved in selective macroautophagy pathways, including mitophagy, aggrephagy, and xenophagy.

## Graphical abstract

**Figure:**
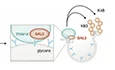

## Introduction

The ubiquitin-proteasome-system (UPS) and the autophagy-lysosomal system are the main degradative processes in eukaryotic cells (Quinet et al, 2020). For a long time, these were considered two strictly separate pathways. Recent evidence, however, has revealed a closer interplay between them, with several complementary and compensatory functions. One common aspect is the conjugation of ubiquitin (Ub) to target structures that must be turned over by the UPS and selective macroautophagy pathways. E3 Ub ligases play a key role in this process. Yet, while a multitude of E3 Ub ligases have been associated with the UPS, only a handful have been linked to selective macroautophagy.

### Ubiquitin and ubiquitination

As the name suggests, Ub is expressed ubiquitously in eukaryotic cells, and functions primarily as a post-translational modifier that regulates protein fate and signaling specificity. Ubiquitination tags intracellular components, such as proteins, protein complexes, organelles or pathogens, with Ub, which triggers substrate turnover to maintain homeostasis. However, ubiquitination is also essential for regulating numerous signaling pathways, including stress response, innate and adaptive immunity and metabolic adaptations by modulating protein activity, localization and signaling (Sun et al, 2020; Oh et al, 2018). In the context of degradation, Ub tags act as a signal that conveys targets such as misfolded proteins to proteasomes, and damaged organelles or pathogens to lysosomes, the latter through endocytosis, endosomal trafficking or autophagy. Although traditionally described as a post-translational protein modification, recent work has demonstrated that Ub can also be conjugated to non-proteinaceous substrates, including bacterial lipopolysaccharides, phospholipids, glycogen and DNA (Otten et al, 2021; Wang et al, 2025; Kelsall et al, 2022; Sakamaki and Mizushima, 2023).

**Figure 1:**
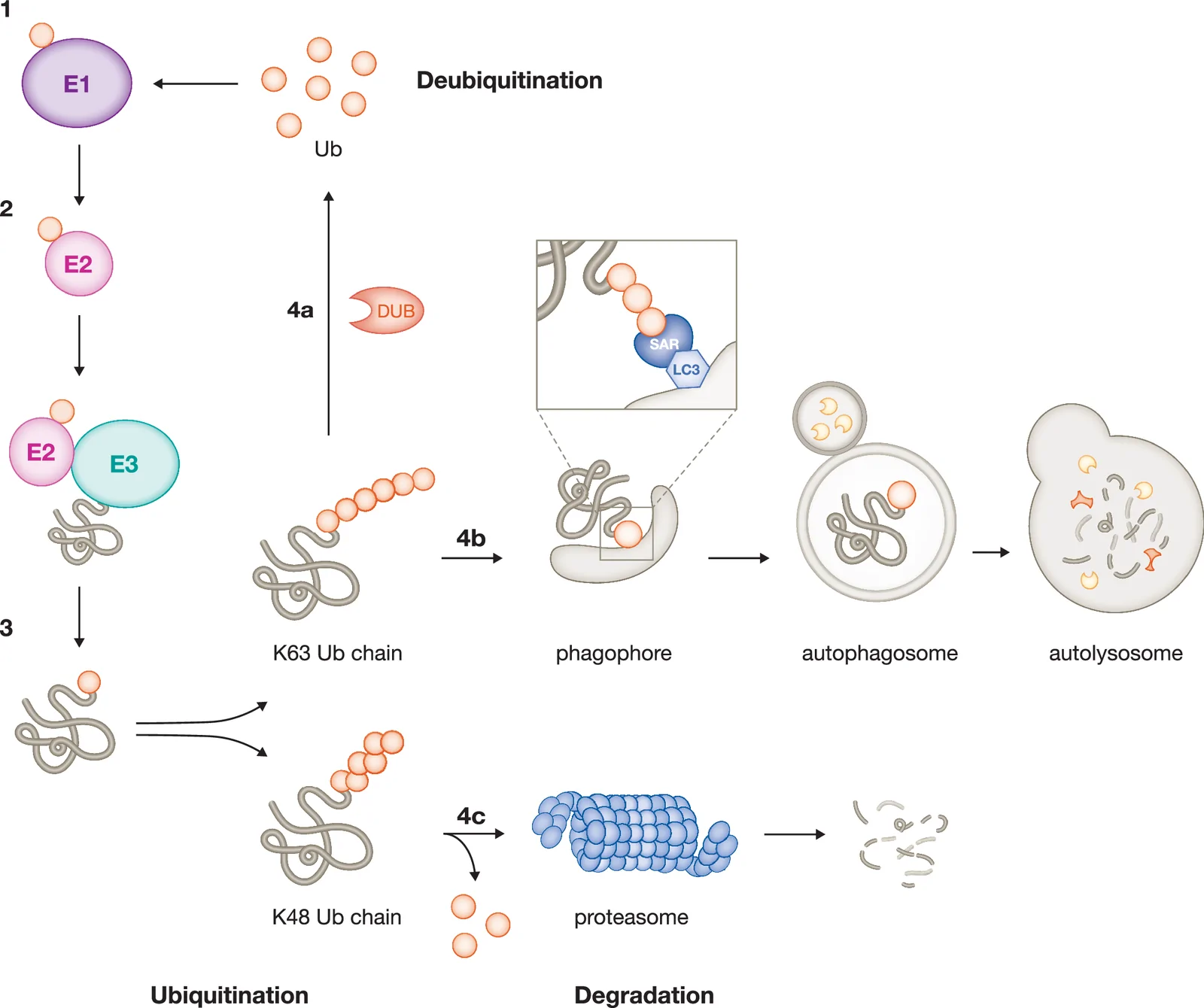
The ubiquitination cascade for targeted proteasomal and/or macroautophagic degradation. A canonical enzymatic cascade ligates Ub to unnecessary or dysfunctional cargos, triggering their turnover. This process is characterized by the sequential activity of E1 activating enzymes (step 1), E2 conjugating enzymes (step 2), and E3 Ub ligating enzymes (step 3). Within poly-Ub chains, Ub is sequentially conjugated via its C-terminus to the N-terminal methionine (M1) or one of its seven internal lysine (K) residues of the previous Ub. Among these linkages, K48-linked polyubiquitination is the principal signal for degradation by the 26S proteasome (step 4c), while K63-linked polyubiquitination conveys substrates to macroautophagic turnover in a process mediated by SARs (step 4b). Cargo selectivity and Ub linkage type selectivity are mainly provided by the E3 Ub ligases. DUBs specifically cleave mono- or poly-Ub chains from substrates, thereby counteracting the E3 activities and maintaining a constant pool of free Ub (step 4a). SAR selective autophagy receptor.

Ub is covalently conjugated to target molecules through an enzymatic cascade (Fig. 1) (Shaid et al, 2013). The Ub tags can consist of either monoubiquitination, multi-monoubiquitination or polyubiquitination of variable length. For the latter, another Ub molecule is sequentially conjugated with its C-terminus to the N-terminal methionine (M1) or one of the seven internal lysine (K) residues of the previous Ub, i.e., K6, K11, K27, K29, K33, K48, or K63 (Ciechanover and Stanhill, 2014; Kravtsova-Ivantsiv et al, 2013). Among these, K48 and K63 are the most abundant ones (Kravtsova-Ivantsiv et al, 2013; Vainshtein and Grumati, 2020). Ub chains can either be homotypic, heterotypic or even branched, and may have additional modifications, such as small ubiquitin-like modifier (SUMO) or neural precursor cell-expressed developmentally downregulated protein 8 (NEDD8), acetylation or phosphorylation, all of which make the Ub tagging distinguishable (Swatek and Komander, 2016). The chain topology appears to be the critical determinant to direct a substrate into a specific degradation route (Shaid et al, 2013). For example, the current simplistic view is that K48-linked polyubiquitination is an important signal for proteasomal degradation, while K63-linked polyubiquitination mostly sends substrates to autophagic turnover (Fig. 1) (Shaid et al, 2013; Vainshtein and Grumati, 2020). These poly-Ub chains are not necessarily homotypic since heterotypic K11- and K48-linked poly-Ub chains also promote proteasomal degradation of a plethora of substrates (Grice et al, 2015).

The canonical enzymatic cascade to ligate Ub to a substrate is characterized by the activity of three different enzymes: E1, E2, and E3 (Fig. 1) (Vainshtein and Grumati, 2020; Cappadocia and Lima, 2017; Zheng and Shabek, 2017; Ebadi et al, 2025). In mammals, the conventional conjugation of Ub moieties to the ε-amino group of a lysine residue in the target protein is initiated by one of two E1 activating enzymes: Ub-like modifier-activating enzyme 1 (UBA1) and UBA6 (Rodrigo et al, 2025) (Fig. 1, step 1). This initial step is followed by a combination of E2 Ub-conjugating enzymes and E3 Ub ligases (Fig. 1, step 2). In the last step, E3 Ub ligases transfer Ub from the E2 conjugating enzyme and conjugate it to the substrate via a covalent bond (Fig. 1, step 3) (Rape, 2018). Although there are only two E1s, there are around 40 E2s and over 600 E3s, each enzyme showing a higher specificity for their target than the one before (Hyer et al, 2018; Oikawa et al, 2020).

E3 Ub ligases are mainly categorized into three families by their type of Ub transfer: the homologous to the E6AP carboxyl terminus (HECT)-type, the new gene (RING)-type, and the RING-in-between-RING (RBR)-type (Dove & Klevit, 2017; Sun et al, 2020). E3 Ub ligases of the HECT family bind E2 Ub-conjugating enzymes with their N-terminal lobe (N-lobe), while the C-terminal lobe (C-lobe) comprises the catalytic amino acid cysteine, where a thioester intermediate of Ub is formed before its transfer to the substrate. RBR E3 Ub ligases create a similar thioester intermediate after recognizing the Ub-binding E2 conjugating enzyme with their RING1 domain, whereas their RING2 contains the catalytic cysteine for the transthioesterification (Jeong et al, 2023; Wang et al, 2023d). Between these two RING domains lays the in-between-RING (IBR) domain, which is required for Ub binding (Jeong et al, 2023; Wang et al, 2023d). In contrast, RING finger E3 Ub ligases can directly transfer Ub to the substrate via their RING or U-box catalytic domain without the need for a thioester intermediate (Jeong et al, 2023). Recently, three additional classes of E3 Ub ligases have been discovered: the RING-Cys-Relay (RCR)-type, the RZ finger-type, and E3–E3-type (Horn-Ghetko and Schulman, 2022). These newly identified classes interact differently with E2 conjugating enzymes and/or possess a different catalytic domain (Horn-Ghetko and Schulman, 2022). Interestingly, pathogenic and symbiotic bacteria can also possess an additional group of E3 Ub ligases encoded by *ipaH* genes, which are important for their pathogenicity (Dranenko et al, 2022). This group was classified separately from the other E3 Ub ligase families as the overall structure is different and only some domains are conserved (Ashida et al, 2010).

Thus, E3 Ub ligases are the main denominator specifying the Ub code, although E2 conjugating enzymes play a role in determining the Ub linkage as well (Yang et al, 2021). The combination of an E3 Ub ligase with different E2 enzymes can determine different Ub modifications (Ferrari et al, 2024). Similarly, deubiquitinases (DUBs) are specific for certain Ub modifications and cleave the mono- or poly-Ub chains from the substrates, thereby counteracting E3 activities (Fig. 1, step 4a) (Clague et al, 2019; Mevissen and Komander, 2017; Takahashi et al, 2020).

### Macroautophagy and the UPS

Autophagy is an umbrella term encompassing all pathways that mediate the delivery and lysosomal turnover of intracellular components; it is essential for cellular homeostasis during both steady-state and stress conditions, as well as generating a pool of nutrients when cells are enduring limited energy supply (Shaid et al, 2013; Vainshtein and Grumati, 2020). There are three major types of autophagy that can be distinguished morphologically. In chaperone-mediated autophagy (CMA), single proteins are translocated into lysosomes. Microautophagy involves the direct engulfment of cargoes by late endosomes and/or lysosomes, while macroautophagy is characterized by the sequestration of the structures targeted to degradation within double-membrane autophagosomes, which then fuse with late endosomes and/or lysosomes (Gatica et al, 2018; Dikic and Elazar, 2018; Vargas et al, 2023).

Macroautophagy is mediated by the ATG proteins, which de novo generate cytoplasmic autophagosomes (Fig. 1). The process is characterized by the nucleation of small membranous cisterna called phagophores or isolation membranes, which elongate by acquiring additional lipids, mostly through direct transport from the endoplasmic reticulum (ER), resulting in double-membrane autophagosomes (Fig. 1, step 4b) (Feng et al, 2014; Nakatogawa, 2020). Approximately 20 ATG proteins compose the core machinery essential for autophagosome biogenesis (Nakatogawa, 2020). This machinery can be subdivided into 6 functional groups: while the ULK kinase complex, the class III phosphatidylinositol 3-kinase complex I and the ATG9A-positive vesicles are central for the nucleation of phagophores, their elongation requires the ATG2-WD repeat domain 45 (WDR45/WIPI4) complex and two non-canonical Ubl conjugation systems that lead to the conjugation of ATG12 and microtubule associated protein 1 light chain 3 (MAP1-LC3)/gamma-aminobutyric acid receptor-associated protein (GABARAP) proteins to ATG5 and mainly phosphatidylethanolamine, respectively (Shaid et al, 2013; Cappadocia and Lima, 2017; Li et al, 2020; Mohan et al, 2024).

Initially, UPS-mediated degradation and macroautophagy were described as separate pathways. Over the years, however, evidence has emerged revealing that these two processes share functional connections with complementary and compensatory attributes (Kocaturk and Gozuacik, 2018; Quinet et al, 2020). For example, one target of the UPS, p53, is a transcription factor for *ATG* genes like *ATG2B*, *ATG4A, ATG4C*, *ATG7*, and *ULK1* (Broz et al, 2013; Wang et al, 2023a). By regulating the levels of p53, the UPS also indirectly controls macroautophagy induction (Quinet et al, 2020). Along the same line, the proteasome can be a substrate for macroautophagy in a process called proteaphagy (Waite et al, 2022). Finally, it has been shown that the proteasome can partially complement the pharmacological or genetic inhibition of macroautophagy (Wang et al, 2013), and conversely, proteasomal inhibition induces macroautophagy (Tannous et al, 2008; Gorbea et al, 2013).

### Selective macroautophagy and the selective autophagy receptors

During nutrient starvation, bulk macroautophagy appears to degrade part of the cytoplasm in a non-selective manner to maintain homeostatic metabolite levels. It is triggered by the activation of AMP-activated protein kinase (AMPK), which detects high levels of AMP and ADP, and activates ULK1 to initiate phagophore nucleation, and/or by the inactivation of mTORC1, which senses cellular nutrient and resource supplies, especially amino acids (Licheva et al, 2021). If nutrients are replete, mTORC1 phosphorylates the ULK kinase complex, inhibiting both activation through AMPK and macroautophagy induction. Additionally, expression of *ATG* and lysosome biogenesis-associated genes is suppressed by the inhibitory phosphorylation of transcription factor EB (TFEB) by mTORC1, reinforcing a block in macroautophagy (Saxton and Sabatini, 2017; Deleyto-Seldas and Efeyan, 2021).

In contrast, selective macroautophagy eliminates specific intracellular structures (Kraft et al, 2010; Shaid et al, 2013), and can occur independent of mTORC1 and AMPK signaling cascades (Saxton and Sabatini, 2017; Adriaenssens et al, 2022). Selective macroautophagy pathways are named based on the cargo that is targeted: aggrephagy (for protein aggregates), ER-phagy (for ER fragments), mitophagy (for mitochondria), lipophagy (for lipid droplets), lysophagy (for lysosomes), ribophagy (for ribosomes), xenophagy for bacteria, viruses and parasites, etc. (Chen et al, 2019; Gubas and Dikic, 2022).

Selective macroautophagy relies on SARs, also referred to as cargo receptors, to ensure accurate recognition of the target (Rogov et al, 2023; Lamark and Johansen, 2021). Current models suggest that SARs orchestrate selective macroautophagy through a coordinated series of events comprising (i) cargo recognition, (ii) recruitment of the ATG machinery, and (iii) guidance of the elongating phagophore membrane around the cargo. SARs bind cargo either directly, as in the case of organelle-resident SARs, or indirectly in the case of soluble SARs, which are recruited to specific cargoes by binding Ub chains via their Ub-binding domains (UBDs) or specific proteins such as galectin 8 (GAL8) (Quinet et al, 2020; Shaid et al, 2013; Gubas and Dikic, 2022; Bell et al, 2021; Kim et al, 2013). SARs then interact with components of the core ATG machinery to induce the nucleation of the phagophore and guarantee the in situ formation of an autophagosome around the cargo (Adriaenssens et al, 2022; Rogov et al, 2023; Lamark and Johansen, 2021; Adriaenssens & Martens, 2025). For example, SARs such as sequestosome 1 (SQSTM1/p62) and nuclear dot protein 52 kDa (NDP52) bind FIP200 and recruit the ULK kinase complex to trigger phagophore formation (Schlütermann et al, 2021; Vargas et al, 2019; Ravenhill et al, 2019). Specific SARs such as NDP52 or optineurin (OPTN) and BCL2 interacting protein 3 like (BNIP3L/NIX) can induce macroautophagy by interacting with TBK1 (and bypassing FIP200 and the ULK kinases) and WIPI2, respectively (Nguyen et al, 2023; Bunker et al, 2023; Adriaenssens et al, 2025). Finally, interactions between LC3 interaction regions (LIRs) in the SARs and LC3/GABARAP proteins in the phagophore membrane allows the selective sequestration of the cargo within autophagosomes (Kirkin and Rogov, 2019). SARs get eventually degraded together with the cargo (Gubas and Dikic, 2022).

Six Ub-dependent SARs have been described, including p62, neighbor of BRCA1 gene 1 (NBR1), TAX1 binding protein 1 (TAX1BP1), NDP52, OPTN and TOLL interacting protein (TOLLIP) (Shaid et al, 2013; Quinet et al, 2020; Adriaenssens et al, 2022). Their activation is a multifactorial process, which includes several post-translational modifications (PTMs), resulting in an enhanced binding affinity for Ub or LC3/GABARAP proteins, but also increases their ability to oligomerize and undergo liquid-liquid phase separation (LLPS) (Farré and Subramani, 2016; Schlütermann et al, 2021; Gubas and Dikic, 2022). For example, phosphorylation of the LIR of OPTN as well as of the UBA domain of p62 by TANK-binding kinase 1 (TBK1) increases their binding affinity to LC3 proteins and Ub chains, respectively (Wild et al, 2011; Matsumoto et al, 2011; Khaminets et al, 2016). Moreover, on the surface of damaged and ubiquitinated mitochondria, recruited NDP52 and OPTN form sheet-like LLPS condensates, facilitating the association of ATG9A-positive vesicles and ultimately of the phagophore membrane (Yang et al, 2024). LLPS of OPTN requires binding of its UBAN domain to poly-Ub chains and is promoted by TBK1-mediated phosphorylation of OPTN (Herrera et al, 2025). Co-condensation of OPTN/poly-Ub with LC3 proteins may help to align ubiquitinated cargo with the autophagosomal membrane. The E3 Ub ligase SMAD ubiquitination regulatory factor-1 (SMURF1) indirectly activates mTORC1 by ubiquitinating and triggering the degradation of its negative regulator phosphatase and tensin homolog deleted on chromosome 10 (PTEN) (Xia et al, 2023). Active mTORC1 phosphorylates p62, increasing its LLPS propensity (Xia et al, 2023). In addition to the Ub-binding SARs, macroautophagy adapter proteins such as autophagy-linked FYVE protein (ALFY) can bind LC3/GABARAP proteins, other components of the ATG machinery and/or SARs, probably to more effectively assemble them into functional complexes (Isakson et al, 2013; Lin et al, 2013; Reinhart et al, 2021).

### Ubiquitination and E3 Ub ligases in mitophagy

Under stress conditions, mitochondrial components released to the cytosol, including mitochondrial DNA, are recognized as damage-associated molecular patterns (DAMPs) and induce immune responses (Dache and Thierry, 2023; Martínez-Reyes and Chandel, 2020; Tiku et al, 2020; Newman and Shadel, 2023). If the damage exceeds a certain threshold, dysfunctional mitochondria are secluded from the mitochondrial network and delivered by mitophagy into lysosomes to be degraded (Dache and Thierry, 2023; Uoselis et al, 2023; Wang et al, 2023c).

**Figure 2:**
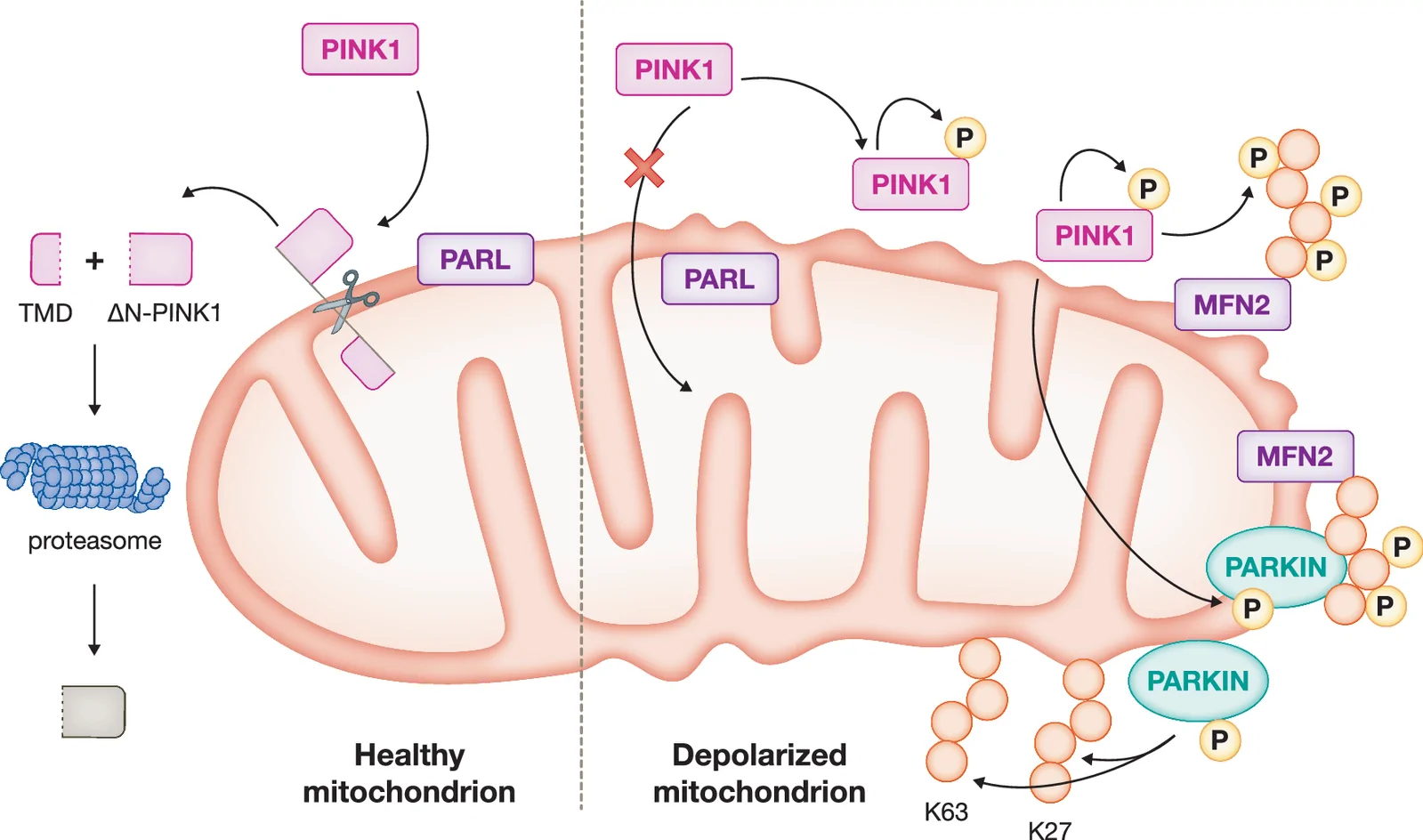
PINK1/PARKIN-mediated mitophagy. Under normal conditions, the PINK1 N-terminal transmembrane domain (TMD) is imported into mitochondria without translocation of the whole protein. The IMM-resident protease PARL then cleaves PINK1’s TMD, generating truncated ΔN-PINK1, which is rapidly degraded by the proteasome. In contrast, mitochondrial depolarization blocks PINK1 import into mitochondria, leading to the accumulation of its full-length form on the OMM, where it phosphorylates preexisting Ub on OMM-resident proteins, such as MFN2. This modification recruits the E3 ligase PARKIN, which is subsequently phosphorylated by PINK1within its Ubl domain. This phosphorylation induces a conformational change that activates PARKIN. Activated PARKIN then interacts with more proteins phosphorylated by PINK1, adding K27- and K63-poly-Ub chains. IMM inner mitochondrial membrane, OMM outer mitochondrial membrane.

Several mechanisms and different SARs have been implicated in mitophagy (Küng et al, 2025). The mitochondrial serine/threonine PTEN-induced putative kinase 1 (PINK1)/E3 Ub ligase PARKIN pathway, is Ub-dependent (Table 1) (Uoselis et al, 2023). Under healthy conditions, PINK1 is targeted to mitochondria, where it is imported and cleaved by the inner mitochondrial membrane-resident PGAM5-associated rhomboid-like serine protease (PARL) into ΔN-PINK1, which is highly unstable and rapidly degraded by the proteasome (Fig. 2) (Heo et al, 2018; Matsuda et al, 2013; Deas et al, 2011). Mitochondrial damage that causes depolarization, i.e., a decrease in membrane potential, blocks PINK1 import into the mitochondria and its processing, leading to its stabilization, accumulation, auto-phosphorylation and activation on the outer mitochondrial membrane (OMM) (Fig. 2) (Matsuda et al, 2013; Chen and Dorn, 2013; Yi et al, 2024). PINK1 then phosphorylates preexisting Ub on OMM-resident proteins such as mitofusin 2 (MFN2), which in turn recruits cytosolic PARKIN through direct binding (Chen and Dorn, 2013); mitochondrial-associated PARKIN Ubl domain is likewise phosphorylated by PINK1 (Fig. 2) (Matsuda et al, 2010; Heo et al, 2015; Kazlauskaite et al, 2014). These modifications induce binding of PARKIN to Ub-chains as well as a conformational change that leads to the activation of PARKIN, providing a positive feedback loop (Table 1 and Fig. 2) (Matsuda et al, 2010; Heo et al, 2015; Kazlauskaite et al, 2014). PARKIN then interacts with more of the proteins previously phosphorylated by PINK1 and, assisted by Ub-conjugating enzyme E2 13 (UBC13), adds K27- and K63-poly-Ub on OMM-resident proteins, including the GTPases MFN1 and MFN2, which have a key role in mitochondrial fusion (Table 1 and Fig. 2) (Dósa and Csizmadia, 2022; Kraft et al, 2010; Khaminets et al, 2016; Heo et al, 2018; Tanaka et al, 2010; Xu et al, 2011; Liu et al, 2022). The enhanced valosin-containing protein (VCP/p97)-mediated proteasomal degradation of MFN proteins increases the number of fission events since their removal prevents mitochondrial re-fusion (Tanaka et al, 2010; Gegg et al, 2010; Gegg and Schapira, 2011; Lamark and Johansen, 2021). The change in mitochondrial dynamics results in a disconnection of damaged mitochondria from the mitochondrial network and their mitophagic degradation (Chen and Dorn, 2013; Tanaka et al, 2010).

**Table 1:** Summary of E3 Ub ligases and types of Ub modifications mediating degradation of various substrates by selective macroautophagy pathways.

Substrate	E3 Ub ligase	Types of Ub chains	New references
Mitochondria	MARCH5	?	(Chen et al, 2017) (Sekine and Youle, 2018) (Di Gregorio et al, 2023)
	MUL1	?	(Gao et al, 2024) (Sekine and Youle, 2018) (Di Gregorio et al, 2023)
	PARKIN (recognizes phosphorylated Ub on mitochondria)	K27-linked polyUb	(Kraft et al, 2010)
		K63-linked polyUb	(Heo et al, 2015) (Dósa and Csizmadia, 2022) (Kraft et al, 2010)
	SMURF1	?	(Orvedahl et al, 2011) (Xu et al, 2025)
	RNF185	K63-linked polyUb	(Di Gregorio et al, 2023) (Tang et al, 2011)
	AMFR (ER-resident)	?	(Fu et al, 2013)
	ARIH1	?	(Villa et al, 2017)
	HUWE1	?	(Di Rita et al, 2018)
synphilin-1	SIAH-1/2	polyUb	(Liani et al, 2004) (Rott et al, 2008)
α-synuclein aggregates	CHIP	?	(Liu et al, 2022) (Shin et al, 2005) (Pratt et al, 2014) (Joshi et al, 2016)
	HOIP	M1-linked polyUb	(Furthmann et al, 2023)
tau aggregates	CHIP	?	(Liu et al, 2022) (Pratt et al, 2014) (Joshi et al, 2016)
	RNF216	K11/K63-linked polyUb	(Zhou et al, 2024)
	HOIP	M1-linked polyUb	(Furthmann et al, 2023)
	?	K48-linked polyUb	(Tan et al, 2008b)
	?	K63-linked polyUb	(Tan et al, 2008b)
TDP43 aggregates	HUWE1 (recognizes previous polyUb chains on aggregates)	?	(Zhou et al, 2023)
	PARKIN	K48-linked polyUb	(Hebron et al, 2013)
		K63-linked polyUb	(Hebron et al, 2013)
	RNF112	?	(Lee et al, 2018)
	HOIP	M1-linked polyUb	(Furthmann et al, 2023)
	CUL2	?	(Uchida et al, 2016)
GAS	CUL1	?	(Yamada et al, 2021)
	PARKIN (recognizes GAL8 on GAS)	K63-linked polyUb	(Cheng et al, 2017)
	? (recognizes S-guanylation on GAS)	K63-linked polyUb	(Ito et al, 2013)
*S. Typhimurium*	RNF213	?	(Otten et al, 2021)
	LRSAM1	K48-linked polyUb	(Huett et al, 2012) (Polajnar et al, 2017)
	ARIH1	K48-linked polyUb	(Polajnar et al, 2017)
	HOIP (recognizes previously appended K63-linked polyUb)	M1-linked polyUb	(Noad et al, 2017) (Van Wijk et al, 2017)
	?	K63-linked polyUb	(Fujita et al, 2013) (Guo et al, 2017) (Polajnar et al, 2017)
*M. tuberculosis*	PARKIN	K63-linked polyUb	(Manzanillo et al, 2013)
	SMURF1	K48-linked polyUb	(Franco et al, 2017)
	TRIM32	mono-Ub	(Romagnoli et al, 2023)
SINV	SMURF1	?	(Orvedahl et al, 2011)
PDCoV	CUL7	K48-linked polyUb	(Ji et al, 2024)
	CHIP	?	(Yang et al, 2023)
	MARCH8	?	(Jiao et al, 2021)
PEDV	MARCH8	?	(Zhai et al, 2023)
*T. gondii*	RNF213	K63-linked polyUb	(Hernandez et al, 2022) (Clough et al, 2016)
Proteasomes	CHIP	K63-linked polyUb	(Choi et al, 2020)
	UBR4	?	(Choi et al, 2020)
	RNF181	?	(Choi et al, 2020)
	?	K11-linked polyUb	(Choi et al, 2020)
	?	K48-linked polyUb	(Choi et al, 2020)
Lysosomes	TRIM16	K63-linked polyUb	(Chauhan et al, 2016) (Vargas et al, 2023) (Zein et al, 2023)
	FBXO27	K48-linked polyUb	(Yoshida et al, 2017) (Vargas et al, 2023) (Zein et al, 2023)
	CUL4A	K48-linked polyUb	(Teranishi et al, 2022)
	FBXO3	?	(Park et al, 2025)
	ITCH	K63-linked polyUb	(Gahlot et al, 2024)
ER	TRIM13	K63-linked polyUb	(Ji et al, 2019)
	CUL3	?	(Wang et al, 2023b)
	AMFR (ER-resident)	?	(González et al, 2023)
Peroxisomes	PEX2	?	(Sargent et al, 2016)
	?	mono-Ub	(Kim et al, 2008)
Inflammasome	TRIM11	?	(Liu et al, 2016b)

NDP52, OPTN, TAX1BP1, and, in part, p62 are redundantly relevant for PINK1/PARKIN-mediated mitophagy: upon recognition of the K27- and K63-poly-ubiquitinated mitochondrial proteins, they recruit the ATG machinery and initiate local mitophagy (Dósa and Csizmadia, 2022; Kraft et al, 2010; Heo et al, 2015; Lamark and Johansen, 2021; Lazarou et al, 2015; Moore and Holzbaur, 2016; Orvedahl et al, 2011). In addition, a different, not mutually exclusive mechanism has been proposed: PINK1-mediated phosphorylation of Ub on OMM-resident proteins that leads to the direct recruitment of NDP52 and OPTN without the requirement of PARKIN (Lazarou et al, 2015). Within this system, PARKIN acts more as an amplifier by increasing the number of K27- and K63-poly-Ub chains on damaged mitochondria (Lazarou et al, 2015). However, this might be cell type-dependent, as another study showed mixed levels of K6, K11 and K48 (Ordureau et al, 2018). Additionally, it has been reported that OPTN and NDP52 association with damaged mitochondria to engage ATG machinery directly depends on PARKIN localization to mitochondria and its activity (Moore & Holzbaur, 2016; Adriaenssens et al, 2024; Imai et al, 2000), although it remains to be established whether PARKIN directly ubiquitinates mitochondrial proteins or amplifies previous Ub signals. Moreover, E3 Ub ligase TRIM5α ubiquitinates TBK1, promoting its interaction with NDP52, p62, and NBR1 upon mitochondrial damage in a PARKIN-dependent and/or -independent manner (Saha et al, 2024). Through the accumulation of SARs on damaged mitochondria, TRIM5α mediates an assembly platform for mitophagy induction (Saha et al, 2024).

PINK1/PARKIN-mediated mitophagy is also relevant for the replication of varicella zoster virus (VZV). The VZV glycoprotein E (gE) induces PARKIN ubiquitination, promotes mitochondrial ROS production, and thereby induces mitophagy. The degradation of mitochondria inhibits the cyclic GMP-AMP synthase (cGAS)-stimulator of interferon genes protein (STING) pathway, which is part of the innate immune system.

Autocrine motility factor receptor (AMFR/GP78) is an ER-resident E3 Ub ligase that ubiquitinates MFN1 and MFN2 on the surface of depolarized mitochondria, inducing mitophagy in a PARKIN-independent manner (Table 1) (Fu et al, 2013). SMURF1 is an additional E3 Ub ligase that can ubiquitinate OMM proteins upon mitochondria depolarization and thereby induces SAR-dependent mitophagy (Table 1). Interestingly, SMURF1 exerts a dual function. Firstly, it ubiquitinates proteins on the OMM via its HECT-domain; these modifications can be recognized by PINK1 to induce mitophagy in a PARKIN-dependent manner (Orvedahl et al, 2011; Xu et al, 2025). Secondly, it has an E3 Ub ligase-independent function that targets damaged mitochondria to the phagophore in what appears to be a SAR-independent mechanism that involves its C2 domain, which may bind phospholipids in the autophagosomal membrane (Orvedahl et al, 2011). Similar to PARKIN, Ariadne RBR E3 ubiquitin protein ligase 1 (ARIH1) appears to be phosphorylated by PINK1 as well and to ubiquitinate OMM-resident proteins on depolarized mitochondria, such as TOM20 and MFN2 (Table 1) (Villa et al, 2017). Overexpression of ARIH1 leads to the increased degradation of p62, suggesting an enhanced mitophagic flux (Villa et al, 2017). HUWE1 ubiquitinates MFN2 during mitophagy triggered by mitochondrial depolarization (Table 1) (Di Rita et al, 2018) in a PINK1/PARKIN-independent manner, suggesting that multiple E3 Ub ligases can prime mitochondria for elimination. This redundancy may be relevant in all cell types that do only possess a subset of E3 Ub ligases or where certain E3 Ub ligases are impaired (Dutta et al, 2025). The RING-type mitochondrial E3 ubiquitin protein ligase 1 (MUL1), membrane-associated ring finger (C3HC4) 5 (MARCH5), and ring finger protein 185 (RNF185) localize to the OMM of damaged or aged mitochondria, and their ubiquitinated substrates may be a target of PINK1 phosphorylation as well (Table 1) (Sekine and Youle, 2018; Di Gregorio et al, 2023; Tang et al, 2011). This notion is supported by the observation that MUL1 and MARCH5 function in mitophagy independently of PARKIN, although MUL1 was also shown to SUMOylate and activate NDP52 during PARKIN-mediated mitophagy (Table 1) (Chen et al, 2017; Gao et al, 2024). In contrast, RNF185 appears to interact with BCL2 19 kDa interacting protein 1 (BNIP1) and tagging it with K63-poly-Ub (Table 1), which recruits p62 and promotes mitophagy (Tang et al, 2011).

While the above-mentioned E3 Ub ligases are relevant to trigger mitophagy, the SCF complex formed by CULLIN1 (CUL1) and F-box and leucine-rich repeat protein 4 (FBXL4) is involved in the suppression of what probably is the main mitophagy pathway, to prevent the disproportionate depletion of mitochondria. This E3 Ub ligase complex ubiquitinates OMM-resident mitophagy SARs BNIP3 and BNIP3L/NIX, leading to their proteasomal turnover and thus negatively regulating BNIP3/BNIP3L-mediated Ub-independent mitophagy (Elcocks et al, 2023; Kulkarni et al, 2024).

### Ubiquitination and E3 Ub ligases in aggrephagy

Conformational changes caused by translation errors or stresses such as oxidation tend to unfold proteins (Shaid et al, 2013; Alberti and Hyman, 2021; Delaby and Lehmann, 2022), which can lead to the formation of protein aggregates (Wen et al, 2023). By the addition of more monomers or oligomers, they become amorphous aggregates or can assemble into higher ordered fibrils (Bauer et al, 2023; Jukovic et al, 2024). In addition, some proteins show an inherent tendency of self-oligomerization (Vainshtein and Grumati, 2020) and ubiquitinated proteins tagged for proteasomal degradation aggregate together after proteasomal block (Kirkin et al, 2009).

Aggrephagy, the selective autophagic clearance of aggregated functional and non-functional proteins, is induced by ubiquitination of aggregates or oligomeric assemblies, and a few E3 Ub ligases are involved. Besides supporting proteins to fold into their correct conformation, HSP70 proteins can also directly interact and recruit the E3 Ub ligase C-terminus of HSC70-interacting protein (CHIP/STUB1), enhancing substrate ubiquitination. Such a mechanism has been shown in CHIP-mediated ubiquitination of tau, α-synuclein, huntingtin and the androgen receptor with an expanded polyQ tract, which are aggregating proteins associated with Alzheimer’s disease (AD), PD, Huntington’s disease and spinal and bulbar muscular atrophy, respectively (Liu et al, 2022; Pratt et al, 2014; Quintana-Gallardo et al, 2019; Joshi et al, 2016). CHIP functions as a molecular switch to direct α-synuclein towards degradation by the UPS or the autophagy-lysosomal system via its tetratricopeptide repeat (TPR) domain and U-box domain, respectively (Table 1) (Shin et al, 2005). CHIP also exerts a dual function in AD: besides ubiquitinating tau and the amyloid-β precursor protein (APP) (Table 1), it also ubiquitinates and mediates proteasomal degradation of BACE1, which leads to a reduced proteolysis of APP (Liu et al, 2022). RNF216 is an RBR-type E3 Ub ligase that ubiquitinates tau with mixed K11/K63-poly-Ub chains (Table 1). Tau then accumulates in an LLPS formed by RNF216 that is targeted for autophagic degradation (Zhou et al, 2024). Other aggregation-prone proteins, such as α-synuclein and transactive response DNA binding protein 43 (TDP-43), might be ubiquitinated by RNF216 through a similar mechanism, but this has not been verified yet (Zhang, 2024).

The E3 Ub ligases seven in absentia homolog 1 (SIAH1) and SIAH2, assisted by E2 conjugating enzyme Ub/ISG15-conjugating enzyme E2 L6 (UBE2L6), introduce poly-Ub modifications on the surface of synuclein alpha interacting protein (SNCAIP/synphilin-1), a protein found in Lewy bodies of several α-synucleinopathies (Wong et al, 2012), and mono-Ub on α-synuclein. The poly-ubiquitination of SNCAIP leads to its simultaneous degradation by the proteasome and macroautophagy, but it remains unclear if monoubiquitination has the same effect (Table 1) (Liani et al, 2004; Rott et al, 2008; Lee et al, 2008). Interestingly, SIAH Ub ligases were found within Lewy bodies of PD patients, indicating a possible role in the pathological development of cytoplasmic inclusion bodies (IBs) (Liani et al, 2004). Indeed, monoubiquitination is an initiation step for the amorphous aggregation of α-synuclein in vitro and in vivo through an unknown mechanism (Rott et al, 2008). However, Ub-specific protease 9X (USP9X) can remove the mono-Ub tags, triggering transport to lysosomes for degradation, suggesting that other (poly)-Ub modifications may be involved in the degradation of α-synuclein by aggrephagy (Rott et al, 2011). It has also been reported that K48- and K63-poly-Ub, appended by so far unknown E3 Ub ligases, support the formation of tau IBs (Table 1) (Tan et al, 2008b). Interestingly, the K63-Ub modification does not only play a role in IB biogenesis, but it is rather responsible for the autophagic clearance of those IBs via p62 (Tan et al, 2008a).

E3 Ub ligases RNF112 and PARKIN polyubiquitinate TDP-43 (Table 1) (Hebron et al, 2013), and aggregated TDP-43 gets primarily degraded via macroautophagy (Lee et al, 2018; Scotter et al, 2014). However, RNF112 attenuates TDP43 aggregate formation, reducing the workload for aggrephagy (Lee et al, 2018). Once a certain density of Ub chains has been reached on the surface of protein aggregates, it appears that the E3 Ub ligase HUWE1 expands and amplifies the Ub modifications (Table 1). This step then recruits VCP/p97 for disaggregation (Zhou et al, 2023). Additionally, it seems that CUL2 E3 Ub ligase ubiquitinates fragmented forms of TDP-43, conveying them to autophagosomal degradation (Table 1) (Urushitani et al, 2010; Uchida et al, 2016).

Aggrephagy relies on the soluble Ub-dependent SARs p62, NBR1, TAX1BP1, NDP52, OPTN, and TOLLIP, although it remains unclear which of them participate in the recognition of the above-described aggregates. These SARs guide the ATG machinery near previously ubiquitinated aggregates and induce their lysosomal destruction (Ma et al, 2022). However, SAR recruitment seems to be not sufficient; their clustering induced by aggregate compaction during fragmentation seems to be one of the mechanisms to initiate autophagosome biogenesis and thus aggrephagy (Mauthe et al, 2025). Clustering of SARs such as p62 and OPTN can also be induced by LLPS-driven condensate formation upon binding to poly-Ub chains (Yang et al, 2024; Herrera et al, 2025; Zaffagnini et al, 2018; Sun et al, 2018). Condensation of p62 at protein aggregates is promoted by its co-condensation with NF-κB essential modulator (NEMO/IKKγ), which, similar to OPTN, has a UBAN domain but lacks the LIR domain (Furthmann et al, 2023). In the absence of NEMO, the formation of the so-called p62 bodies and the subsequent recruitment of the ATG machinery in a M1-linked poly-Ub-dependent manner are impaired, resulting in a widespread mixed brain proteinopathy characterized by the presence of α-synuclein, tau and TDP43 aggregates in a patient with a mutation in the *NEMO* gene (Furthmann et al, 2023) (Table 1). Interestingly, mutations in p62 and OPTN, affecting their LIR or UBA(N) domains, have been associated with neurodegenerative diseases such as amyotrophic lateral sclerosis (ALS), underlining the physiological importance of SARs in aggrephagy (Zhao et al, 2024; Deng et al, 2017; Rubino et al, 2012; Ayaki et al, 2018).

p62 associates to protein aggregates principally by binding to K63-, K48-, and especially M1-poly-Ub linkages (Furthmann et al, 2023; Wurzer et al, 2015). This SAR self-oligomerizes to form helical filaments via its Phox1 and Bem1p (PB1) domain and/or undergoes LLPS to condensate the cargo, initiate phagophore nucleation and stabilize the binding with both LC3/GABARAP proteins and ubiquitinated proteins, which is supported by binding of p62 to M1-linked poly-Ub (Furthmann et al, 2023; Wurzer et al, 2015; Bjørkøy et al, 2005; Zhang et al, 2018; Kumar et al, 2022; Bin et al, 2023; Jakobi et al, 2020). p62 itself gets ubiquitinated in the presence of E2 conjugating enzymes UBE2D2/UBE2D3; among the contributing E3 Ub ligase are CUL3, tripartite motif 21 (TRIM21), RNF166, NEDD4, and RNF26 (Peng et al, 2017; Lee et al, 2017; Pan et al, 2016; Heath et al, 2016; Lin et al, 2017; Jongsma et al, 2016). While the removal of K48- and K63-poly-Ub tags appended to the PB1 domain by the DUB USP13 facilitates the self-oligomerization of p62 (Bin et al, 2023), phosphorylation of its UBA domain and LLPS-mediated formation aided by SMURF1 increase the binding affinity for poly-Ub, promoting the targeting of ubiquitinated proteins to the p62-positive aggregates/and turnover by aggrephagy (Schlütermann et al, 2021; Matsumoto et al, 2011; Xia et al, 2023). TAX1BP1 is also recruited to sites of condensation with the help of NBR1, and it triggers the recruitment of FIP200 (Schlütermann et al, 2021; Turco et al, 2021). Although p62, NBR1, and TAX1BP1 initiate aggrephagy of LLPS aggregates, which are preferentially degraded by selective macroautophagy, TRiC subunit chaperonin-containing TCP1 subunit 2 (CCT2) appears to promote aggrephagy clearance of solid aggregates in an Ub-independent manner (Ma et al, 2022; Zhao et al, 2024).

### Ubiquitination and E3 Ub ligases in xenophagy

Selective macroautophagy plays an important role in innate immunity as xenophagy can eliminate cytosolic bacteria, viruses, fungi and even whole parasites (Khaminets et al, 2016). In contrast to other selective pathways, such as mitophagy and aggrephagy, xenophagy is not contributing to the cell metabolism, but guarantees cell survival by removing pathogens and combating infections (Dósa and Csizmadia, 2022). One of the most important steps is the recognition of cell-foreign antigens and thus distinguishing them from endogenous components, to specifically remove only the intruders (Khaminets et al, 2016).

**Figure 3:**
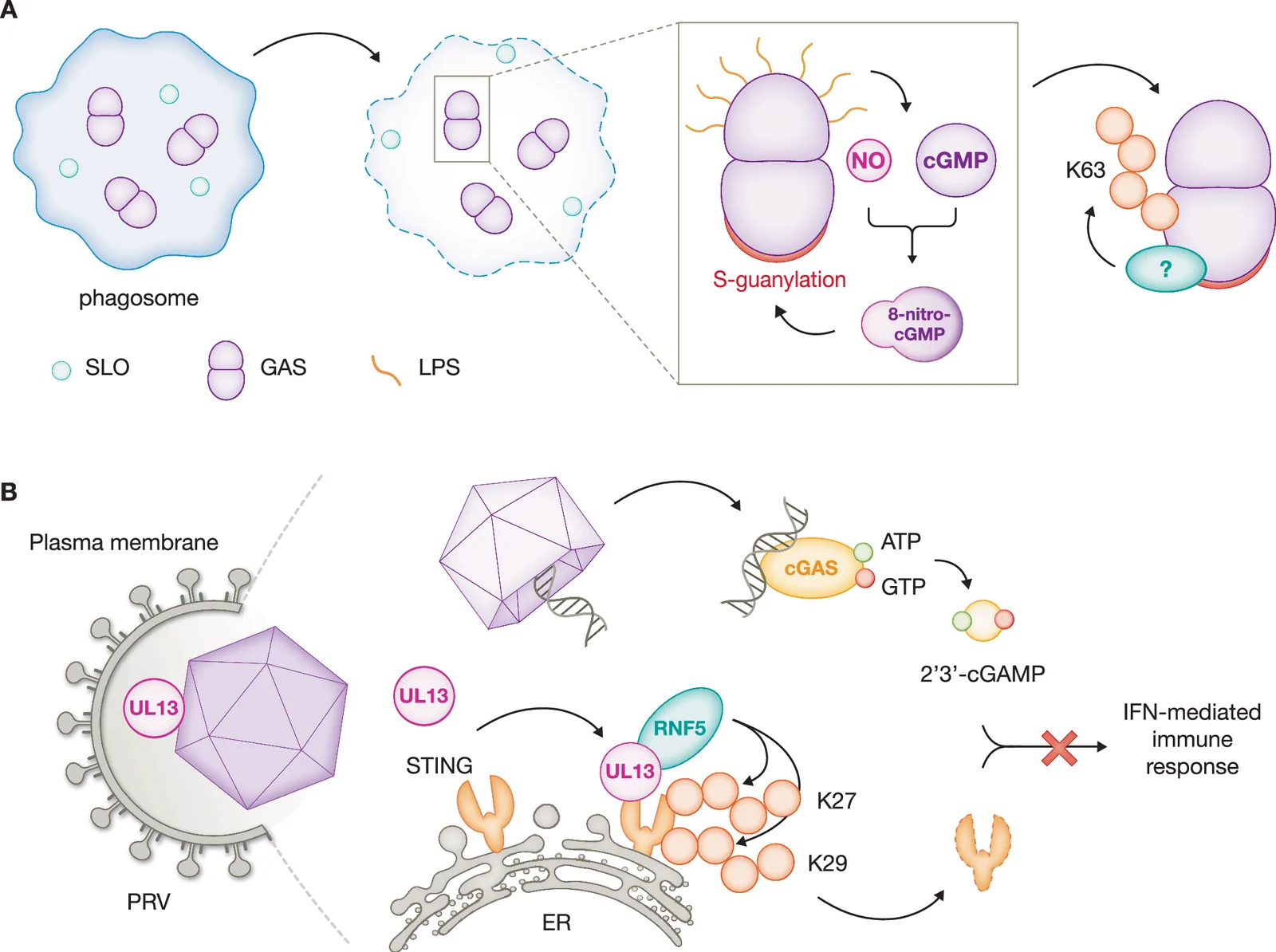
Examples of pathogen-host interactions during xenophagy. (**A**) GAS secretes SLO to damage the phagosomal membrane upon entering macrophages, enabling its infiltration of the host cell cytoplasm. During GAS infection, NO promotes cGMP nitration into 8-nitro-cGMP, which can alter the surface of cytoplasmic GAS via S-guanylation. This induces K63-poly-ubiquitination on the bacterial surface by an unknown E3 Ub ligase and subsequently promotes xenophagy. (**B**) cGAS recognizes viral dsDNA and produces 2’3’-cGAMP, which binds STING and induces an IFN-mediated immune response. PRV disassembly releases the viral protein UL13 is released in the host cell cytoplasm. UL13 directly binds STING and recruits the E3 Ub ligase RNF5, which ubiquitinates STING via K27- and K29-Ub-linkages. This modification leads to the degradation of STING and inhibits STING-mediated immune responses. GAS group A *Streptococcus*, SLO streptolysin O, LPS lipopolysaccharide, NO nitric oxide, PRV pseudorabies virus.

It is a constant evolutionary competition between host cell defense mechanisms and xenogeneic bypass strategies. *Streptococcus pyogenes*, also called group A Streptococcus (GAS), secretes streptolysin O (SLO) to damage either the phagosomal membrane upon phagocytosis by macrophages or the endosomal membrane of non-phagocytic cells, enabling infiltration of the host cell cytoplasm (Fig. 3A) (Nakagawa et al, 2004; Hancz et al, 2019; Bergsten and Nizet, 2024). During GAS infection, nitric oxide (NO) promotes cyclic guanosine monophosphate (cGMP) nitration into 8-nitroguanosine 3′,5′-cyclic monophosphate (8-nitro-cGMP), a macroautophagy inducer (Fig. 3A) (Ito et al, 2013). Endogenous 8-nitro-cGMP can alter the surface of cytoplasmic GAS via S-guanylation, which in turn induces K63-poly-ubiquitination on the bacterial surface (Table 1; Fig. 3A) (Ito et al, 2013). The E3 Ub ligase(s) carrying out this modification remain unknown. In addition to S-guanylation, GAL8 covers the GAS surface or the remnants of endosomal structures after intracellular invasion in epithelial cells, which recruits PARKIN for subsequent K63-linked polyubiquitination (Table 1) (Wang et al, 2020; Cheng et al, 2017; Poole et al, 2018). *N*-acetylglucosamine side chains in the carbohydrates present on the surface of GAS can be recognized by substrate-recognition subunit F-Box only protein 2 (FBXO2), and this promotes the subsequent ubiquitination of the GAS surface by CUL1 (Table 1) (Yamada et al, 2021). Cytoplasmic GAS are also decorated with p62, suggesting lysosomal targeting (O’Neill et al, 2016). However, some GAS strains secrete the cysteine protease streptopain (SpeB) into the cytoplasm, which degrades p62 as well as other SARs like NBR1 and NDP52, thereby counteracting the induction of xenophagy (Barnett et al, 2013).

The E3 Ub ligase leucine-rich repeat and sterile alpha motif containing 1 (LRSAM1) binds Gram-negative *Salmonella Typhimurium* via its LRR-domain and ubiquitinates this bacterium with K48-linked poly-Ub-chains upon its exit from phagosomes (Table 1) (Huett et al, 2012; Polajnar et al, 2017). Likewise, ARIH1 decorates cytosolic *S. Typhimurium* with K48-poly-Ub (Polajnar et al, 2017) (Table 1). It appears that LRSAM1 and ARIH1 are part of a tightly regulated network with compensatory function, which ensures ubiquitination of cytosolic bacteria even upon loss of individual ligases (Polajnar et al, 2017). *S. Typhimurium* was also found to be decorated with K63-poly-Ub chains, however, these are likely appended by other E3 Ub ligases than LRSAM1, since LRSAM1 is known to preferentially assemble K6-, K27-, K29-, and K48-linked poly-Ub chains (Table 1) (Fujita et al, 2013; Guo et al, 2017; Polajnar et al, 2017). The E3 Ub ligase RNF213 restricts bacterial proliferation by ubiquitinating the lipid A of lipopolysaccharides (LPS) of *S. Typhimurium* (Table 1) (Otten et al, 2021). RNF213 also responds to Gram-positive bacteria, but the substrate remains unclear since those have no LPS (Crespillo-Casado et al, 2024). RNF213-mediated self- and LPS-ubiquitination is not carried out via its RING domain but instead by its RZ finger in the C-terminal lobe, which represents a new Ub E3 ligase motif (Otten et al, 2021; Horn-Ghetko and Schulman, 2022; Crespillo-Casado et al, 2024). This step is essential for the recruitment of the LUBAC subunit HOIP and thus M1-poly-ubiquitination (Otten et al, 2021). HOIP recognizes preexisting K63-linked Ub chains appended by other E3 Ub ligases on *S. Typhimurium* via its double NZF (dNZF) domain, directing the LUBAC complex to the bacterial surface and enabling subsequent poly-ubiquitination via M1 linkage (Table 1) (Noad et al, 2017; Van Wijk et al, 2017). LUBAC may possibly compensate for the loss of E3 Ub ligases by activating NF-κB signaling (Polajnar et al, 2017). For the induction of xenophagy upon an *S. Typhimurium* infection, it seems crucial that NDP52 binds LC3C, since the other members of the LC3/GABARAP protein family could not sufficiently execute bacterial clearance in the absence of LC3C (von Muhlinen et al, 2012). In contrast, OPTN, which is also recruited onto the bacterium, binds LC3A and LC3B, but it is unknown if these two isoforms are crucial for the xenophagic removal of bacteria (Wild et al, 2011). Interestingly, OPTN, but not p62, and NDP52, requires LUBAC and therefore M1-Ub chains for its recruitment to cytoplasmic *S. Typhimurium* (Noad et al, 2017), while LRSAM1 reinforces NDP52 and p62 localization on the bacterial surface (Huett et al, 2012). This supports the notion of the existence of compensatory pathways for xenophagy that involve different E3 Ub ligases and SARs, which may be pathogen- and cell type-specific (Noad et al, 2017; Clough et al, 2016).

PARKIN, SMURF1, and TRIM32 were shown to conjugate K63-poly-Ub, K48-poly-Ub and mono-Ub modifications, respectively, to *Mycobacterium tuberculosis* (Table 1) (Manzanillo et al, 2013; Romagnoli et al, 2023; Franco et al, 2017). *M. tuberculosis* covered mainly with K63-Ub modifications co-localizes with NBR1, NDP52, phosphorylated TBK1 and p62, which has a high affinity for K63-poly-Ub chains (Tan et al, 2008a; O’Neill et al, 2016; Manzanillo et al, 2013; Franco et al, 2017). Additionally, and opposite to GAS, the cGAS-STING axis is important for autophagosomal clearance of *M. tuberculosis* via NDP52 through an unknown mechanism (Watson et al, 2015). Interestingly, *Listeria monocytogenes* and several other bacteria produce the STING agonist c-di-AMP, which probably can counteract this STING-mediated autophagic degradation (Watson et al, 2015; Stülke and Krüger, 2020). Other bacteria have evolved different strategies to avoid xenophagy. For example, *Shigella flexneri* was shown to specifically antagonize LUBAC-mediated recruitment of OPTN and more generally xenophagy by ubiquitinating and directing LUBAC to proteasomal degradation via the bacterial E3 Ub ligase IpaH1.4 (Noad et al, 2017).

The study of *S. Typhimurium* infection has revealed that, in addition to be recognized by SARs, Ub-coated bacteria directly bind ATG16L1 via an interaction between its WD β-propeller and Ub (Fujita et al, 2013). ATG16L1 directly binds FIP200, initiating downstream LC3 protein recruitment and lipidation for lysosomal degradation (Fujita et al, 2013). This observation suggests that substrate ubiquitination does not induce local autophagosome biogenesis exclusively via SARs, but it may also involve an interaction with ATG components, providing plasticity to the cargo recognition system of selective macroautophagy.

Most of the studies focus on the lysosomal elimination of bacteria; the study of virophagy is more complex. Upon cell entry, viruses disassemble into single proteins and DNA or RNA, making it difficult to track them. Moreover, viruses often exhaust different strategies that enable effective circumvention or active block of host defense mechanisms. For example, by binding host BECN1 via infected cell protein 34.5 (ICP34.5), herpes simplex virus type 1 (HSV-1) impedes ICP34.5 lysosomal degradation (Orvedahl et al, 2007; Tallóczy et al, 2006). Additionally, the serine/threonine-protein kinase and tegument protein unique long region 13 (UL13) of pseudorabies virus (PRV) directly binds STING and recruits the E3 Ub ligase RNF5 (Fig. 3B). RNF5 then ubiquitinates STING via K27- and K29-Ub-linkages that lead to the degradation of STING and inhibit STING-mediated antiviral signaling (Fig. 3B) (Kong et al, 2022). UL21 from PRV but also HSV-1 counteracts the host cell’s cGAS-STING pathway by recruiting Ub protein ligase E3C (UBE3C) and thereby mediating K27-linked polyubiquitination of cGAS. This Ub modification is recognized by TOLLIP, which induces the lysosomal degradation of cGAS (Ma et al, 2023).

There is some evidence indicating a role of xenophagy in counteracting viral infections by recognizing specific viral proteins. During a Sindbis virus (SIN) infection, SMURF1 interacts with the SIN capsid, and targets it for autophagosomal degradation (Table 1), indirectly shown by co-localization with members of the LC3 protein family. However, this is independent of p62 (Orvedahl et al, 2011). Accordingly, while the entire HSV-1 virions egress the host cell cytoplasm in *smurf1*^*−/−*^ MEFs, a partial population of the virions is targeted to autolysosomal structures in wild-type cells (Orvedahl et al, 2011).

During porcine deltacoronavirus (PDCoV) infection, F-box and WD repeat domain containing 8 (FBXW8) recognizes the nucleocapsid (N) protein and, by recruiting the E3 RING-ligase CUL7, mediates its K48-linked polyubiquitination (Table 1). This leads to suppressed replication of PDCoV, suggesting a xenophagic turnover of the N protein (Ji et al, 2024). The host adapter proteins polyadenylate-binding protein 4 (PABPC4) and PGAM family member 5 (PGAM5) appear to also recognize the PDCoV N protein and facilitate binding of the E3 Ub ligases MARCH8 and CHIP, respectively (Table 1). The resulting Ub code, which remains to be deciphered, recruits NDP52 and p62, leading to lysosomal degradation of the N protein (Jiao et al, 2021; Yang et al, 2023). The N protein of the porcine epidemic diarrhea virus (PEDV) is likewise degraded in lysosomes via the MARCH8/NDP52-axis, but this requires the restriction factor heterogeneous nuclear ribonucleoprotein A1 (HNRNPA1), which directly binds and bridges the PEDV N protein with MARCH8 (Table 1) (Zhai et al, 2023). Overall, MARCH8 possesses a large antiviral spectrum against the cytoplasmic tails of several envelope glycoproteins from different virus families. For example, it ubiquitinates rabies virus G protein, SARS-CoV and SARS-CoV-2 spike protein, and Ross River virus E2 proteins (Zhang et al, 2022). However, it remains to be investigated whether the modification of these proteins by MARCH8 targets them to proteasomal or lysosomal degradation.

Intracellular parasites and their recognition by E3 Ub ligases for xenophagic degradation have not been studied intensively yet either. Nonetheless, it has been reported that *Toxoplasma gondii*-containing vacuoles get ubiquitinated with linear and K63-poly-Ub chains in an RNF213-mediated process (Table 1) (Hernandez et al, 2022; Clough et al, 2016). *Trypanosoma cruzi* is decorated with Ub and co-localizes with NDP52 and p62 (Casassa et al, 2019). Upon downregulation of Ub by siRNA, the infection rate of *Leishmania major* increases significantly, indicating the importance of ubiquitination for the removal of intracellular parasites (Frank et al, 2015). In *Caenorhabditis elegans*, the Skp1−Cul−F-box protein (SCF) E3 Ub ligase complexes formed by Skp1, one of multiple cullins, and a variable F-box protein for substrate recognition, play an important role in the turnover of a wide range of substrates (Bakowski et al, 2014; Nayak et al, 2002). Knockdown of Skp1-related (*skr*) genes *skr-3* and *skr-5*, as well *as cul-6* in this worm, increased the pathogen load of *Nematocita parisii* in vivo, suggesting that SCF E3 Ub ligase complexes play an important role in limiting *N. parisii* growth by mediating xenophagy (Bakowski et al, 2014). However, since there is a large number of SCF complexes in humans (Bakowski et al, 2014), the translation of this finding is not straightforward.

### Ubiquitination and E3 Ub ligases in other selective types of macroautophagy

To maximize protein degradation capacity, the UPS and autophagy-lysosomal system machineries must run simultaneously and be coordinated. Under proteotoxic stress or proteasome impairment, it becomes necessary for macroautophagy to take over the workload of proteasomes to ensure cellular homeostasis. To promote macroautophagy activity, impaired proteasomes can selectively be degraded by macroautophagy through a process called proteaphagy (Quinet et al, 2020). Upon chemical proteasome inhibition with MG132, the 26S particle gets extensively ubiquitinated. One of the responsible E3 Ub ligases is CHIP, which mainly conjugates K63-Ub chains to the proteasome (Choi et al, 2020), but other ones, such as UBE3A, ubiquitin protein ligase E3 component n-recognin 4 (UBR4), and RNF181, also appear to contribute (Table 1) (Choi et al, 2020). Ubiquitination induces the transport of the impaired proteasome to aggresomes via histone deacetylase 6 (HDAC6), an Ub receptor bridging ubiquitinated cargo to the motor protein dynein (Choi et al, 2020). Aggresomes are insoluble fractions within the cytoplasm and facilitate autophagic degradation by being a collection point for unwanted material that must be turned over (Johnston et al, 1998).

In yeast, the two E3 Ub ligases Rsp5 and Hul5 ubiquitinate aggresome-localized proteasomes that have been exported from the nucleus after ubiquitination by San1 (Marshall and Vierstra, 2022). At aggresomes, the ubiquitinated dysfunctional proteasomes are recognized by p62, which induces their autophagic degradation via binding to LC3B (Marshall et al, 2015; Choi et al, 2020; Lopez-Reyes et al, 2021). Interestingly, it seems that even under non-selective macroautophagy triggered by starvation, there is some disassembling of the proteasome into individual CPs and RPs that leads to partial lysosomal turnover to shift the degradative equilibrium towards the less energy-consuming macroautophagy, which also rapidly degrades greater amounts of proteins (Quinet et al, 2020).

Lysosomal membranes can get disrupted by the engulfed material, e.g., β-amyloid aggregates or uric acid crystals in acute hyperuricaemic nephropathy, which can cause harmful consequences by hydrolases leaking from damaged lysosomes (Maejima et al, 2013). Central for lysophagy flux is TAX1BP1, which triggers this pathway by binding TBK1 and FIP200 (Eapen et al, 2021; Kakuda et al, 2023).

**Figure 4:**
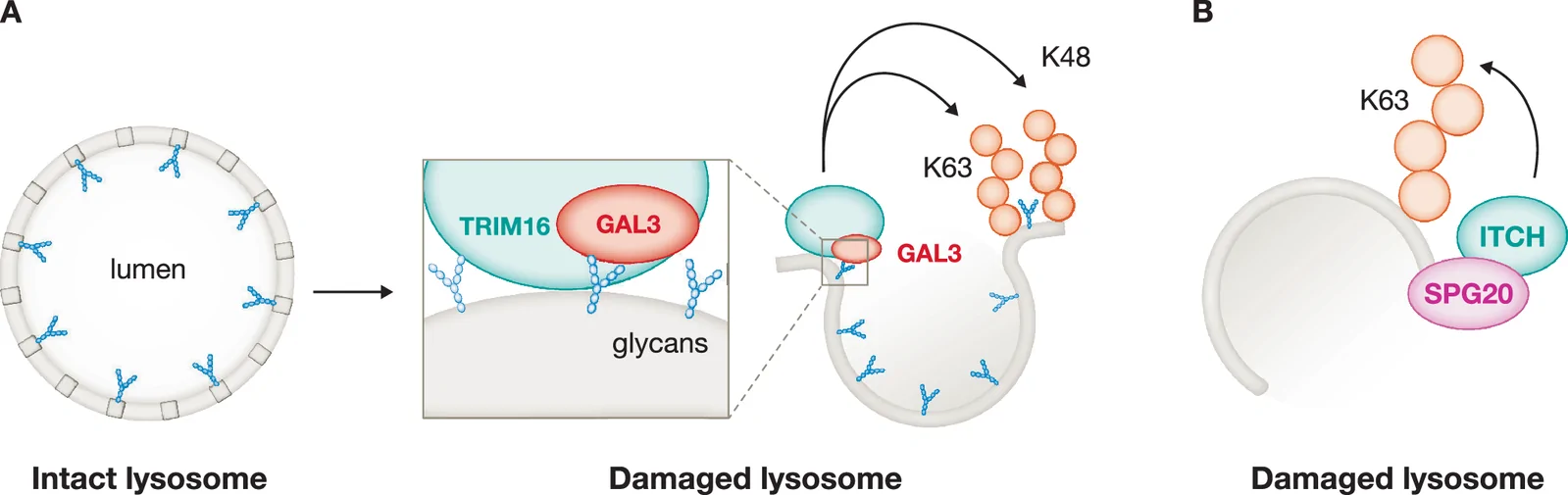
Players recognize lysosomal damage and modulate downstream responses. (**A**) Luminally oriented glycans get exposed on the surface of lysosomes upon disruption of their limiting membrane. GAL3 directly binds those glycans and recruits the E3 Ub ligase TRIM16. While K63-poly-Ub chains appear to be part of an initial response to lysosomal damage, K48 modifications seem to occur at a later stage. (**B**) ITCH is recruited to damaged lysosomes by the lipid perturbation-sensing protein SPG20 and ligates K63-poly-Ub to initiate lysophagy.

Lysosomal damage can be recognized by GAL3, which interacts with the luminally oriented glycans that get exposed upon lysosomal membrane disruption (Fig. 4A) (Vargas et al, 2023). GAL3 directly binds and recruits the E3 Ub ligase TRIM16 in the presence of ULK1 (Table 1; Fig. 4A) or the FBXO27 subunit of the SCF E3 Ub ligase complex (Table 1) (Chauhan et al, 2016; Yoshida et al, 2017). The Ub code pattern created by these E3 Ub ligases seems to go through a specific time course, with K63-poly-Ub chains found as a first response to lysosomal damage and K48 modifications occurring at a later stage (Fig. 4A) (Vargas et al, 2023; Zein et al, 2025). K48-poly-Ub chains are generated by FBXO27 with the support of Ub-conjugating enzyme E2 QL1 (UBE2QL1) (Yoshida et al, 2017; Koerver et al, 2019). Interestingly, VCP/p97 and the DUB YOD1 are recruited after p62 to partially remove K48 modifications from lysosomes and to shift lysosomes from endolysosomal damage response towards lysophagy (Papadopoulos et al, 2016). This step seems to be crucial for correct lysophagy, since accumulation of lysosomes covered by K48-poly-Ub chains are detected in multisystem proteinopathies such as ALS and IB myopathy (Vargas et al, 2023). Among the proteins that have been found ubiquitinated in damaged lysosomes were VAMP3, VAMP7, LAMP1, LAMP2, and transmembrane protein 192 (TMEM192) (Yoshida et al, 2017). It appears that lysophagy can be induced by several, very likely redundant, pathways (Zhu et al, 2020), and multiple other E3 Ub ligases have been identified. CUL4A, in a complex with damage-specific DNA binding protein 1 (DDB1) and WD repeat and FYVE domain containing 1 (WDFY1), specifically tags LAMP2 and GAL3 with K48-linked poly-Ub (Table 1) (Teranishi et al, 2022). WDFY1 and partially DDB1 are responsible for localizing CUL4A to damaged lysosomes (Teranishi et al, 2022). Simultaneously, E3 Ub ligase F-box protein 3 (FBXO3) interacts and ubiquitinates TMEM192 (Table 1) (Park et al, 2025). Another E3 Ub ligase relevant for lysophagy is the Itchy E3 ubiquitin protein ligase (ITCH), which is recruited to damaged lysosomes by the lipid perturbation-sensing protein SPG20, and that ligates K63-poly-Ub to initiate lysophagy (Table 1; Fig. 4B) (Gahlot et al, 2024).

ER-phagy selectively degrades ER fragments to remove damaged ER and reduce ER size after stress conditions (Li et al, 2021) and during starvation (Mo et al, 2020). While ER-phagy is mainly mediated by Ub-independent mechanisms, a few studies suggest the involvement of Ub. The ER-resident members of the family with sequence similarity 134 (FAM134) protein family are Ub-independent SARs relevant for ER-phagy and ER morphology control, and thereby essential for cell homeostasis (Khaminets et al, 2015). It has been shown that ubiquitination of the reticulon homology domain of FAM134B leads to its clustering, which in turn stimulates FAM134B binding to lipidated LC3B (González et al, 2023). This Ub modification is mediated by E3 Ub ligase AMFR/GP78 (Table 1) and ultimately leads to an increase in ER-phagy flux (González et al, 2023). Interestingly, as for mitophagy, PINK1 also contributes to the clearance of ER parts by binding the kelch-like ECH-associated protein 1 (KEAP1) subunit of the KEAP1/CUL3 E3 Ub ligase complex and thereby localizing CUL3 to the surface of the damaged ER (Table 1). CUL3 ubiquitinates the ER-phagy receptor reticulon-like1 (Rtnl1) in *Drosophila*, an orthologue of the mammalian ER-phagy SAR reticulon-3 (RTN3), which then interacts with Atg8a, promoting ER-phagy (Wang et al, 2023b). TRIM13 is an ER-resident E3 Ub ligase that ubiquitinates itself with K63-poly-Ub chains (Table 1) when ER-resident proteins are N-terminally arginylated under ER-stress, leading to p62 recruitment and ER-phagy induction (Ji et al, 2019). Association of p62 with the ER during the metabolism of xenobiotics and subsequent proliferation of hepatic ER indicates the involvement of Ub in ER-phagy as well (Yang et al, 2016).

Ribophagy in yeast works through an opposite mechanism. While the E3 Ub ligase Ltn1/Rkr1 associates with the 60S ribosome, inhibiting the selective degradation of the large ribosomal subunit by ubiquitinating Rpl25 with a yet to be determined Ub code, the reversion of this modification by the DUB complex Ubp3-Bre5 and starvation-induced decrease of Ltn1 enables ribophagy (Ossareh-Nazari et al, 2014). Nuclear FMR1-interacting protein 1 (NUFIP1) is a mammalian SAR that binds ribosomes and LC3B under nutrient-depletion (Wyant et al, 2018). Which determinant on ribosomes is detected by NUFIP1 and whether NUFIP1 is an Ub-dependent SAR remains to be revealed, but a speculative idea would be that, since NUFIP1 acts upon starvation, there possibly is a mechanistic connection between this SAR and the system described in yeast (Box 1).

A category of selective macroautophagy cargoes that is often overlooked is single proteins and small protein complexes. It remains unclear why their turnover is executed through macroautophagy and not the proteasome, but this may be due to their assembly in complexes or condensates. One example is absent in melanoma 2 (AIM2), a subunit of the inflammasome that is important for cytosolic signaling and the maturation of proinflammatory cytokines (Cong et al, 2020). Upon cell infection by a DNA virus, TRIM11 downregulates the activity of the AIM2 inflammasome, which is normally activated by both AIM2 binding to viral DNA, and by self-ubiquitination (Table 1). This in turn mediates p62 recruitment and the selective degradation of AIM2 by macroautophagy (Liu et al, 2016a).

Notably, single proteins can be sequestered by autophagosomes upon monoubiquitination as well (Kim et al, 2008). However, this was tested on non-native cellular proteins, and the relevance of tagging small proteins with mono-Ub for selective macroautophagy needs to be validated with endogenous substrates. Interestingly, monoubiquitination seems to be crucial for pexophagy as well (Table 1) (Kim et al, 2008). One relevant E3 Ub ligase is peroxisomal biogenesis factor 2 (PEX2), which ubiquitinates the peroxisomal surface proteins PEX5 and peroxiredoxin 5 (PRDX5), leading to the recruitment of NBR1 and macroautophagic sequestration (Table 1) (Sargent et al, 2016). In budding yeast, Pex2 mediates polyubiquitination, whereas Pex12 enables monoubiquitination of Pex5 in a Pex4-dependent manner (Platta et al, 2009).

Box 1 In need of answers

- How do E3 Ub ligases recognize the substrate that must be removed by the different selective macroautophagy pathways?
- Why are there so many E3 Ub ligases involved in certain types of selective macroautophagy?
- Why are different types of Ub chains involved in the macroautophagic turnover? What is the function of heterotypic Ub chains in this context?
- How is cargo ubiquitination and SAR activation orchestrated by E3 Ub ligases? Are these steps initiated simultaneously or sequentially?

## Conclusions

Most of the studies investigating Ub-dependent selective macroautophagy focus on the regulation, mechanism and physiological impacts of the degradation of specific cargoes. The Ub code and recognition of the tagged target by the proteasome or macroautophagy is essential for cell survival, and the specifics of how Ub moieties are attached or removed ultimately decide the fate of the cargo. However, only a few studies have associated specific E3 Ub ligases and distinct Ub codes with a cargo or a subset of cargoes. What clearly emerged in our review is that our knowledge about the identity of Ub E3 ligases, which can direct substrates into lysosomal degradation, remains largely incomplete.

Additional important questions to be addressed are, for example, how SAR activation and cargo ubiquitination by specific E3 Ub ligases are orchestrated, and whether these steps are initiated simultaneously or sequentially. It would also be valuable to analyze how impairment of a specific selective macroautophagy contributes to disease progression, since some E3 Ub ligases and SARs are important for several pathways. Thus, future studies on E3 Ub ligases involved in selective macroautophagy will be relevant in a medical context with the aim of redirecting them to specific substrates for treatment. For example, understanding how mitophagy induction is regulated by E3 Ub ligases may be important to delay aging processes or restrict the symptoms of neurodegenerative disorders and myopathies that are characterized by the accumulation of damaged mitochondria (Gherardi et al, 2025).

Like proteolysis targeting chimeras (PROTACs), which catalyze K48-poly-Ub chains for proteasomal degradation by controlling the association of an E3 Ub ligase to a specific substrate (Li & Crews, 2022; Němec et al, 2022), autophagy-targeting chimeras (AUTACs) induce K63-Ub linkages on determined structures and direct them to lysosomal turnover through a conceptually similar strategy (Zhou et al, 2025; Takahashi et al, 2019). AUTACs are particularly advantageous to eliminate substrates that cannot be cleared by the proteasome, and a better understanding of the molecular principles of E3 Ub ligases mediating selective macroautophagy could broaden the applicability of AUTACs to numerous diseases.

## Supplementary information

Peer Review File
