# Supplementary material for: The role of E3 ubiquitin ligases in selective types of macroautophagy
Source: EMBO Rep. 2026 Jul 23;27(17):4950–68. doi: 10.1038/s44319-026-00887-1 (PMC13554234; doi:10.1038/s44319-026-00887-1)
Supplement: Supplementary file 1 [file 44319_2026_887_MOESM1_ESM.pdf]

EMBO reports

The role of E3 ubiquitin ligases in selective types of macroautophagy

Monja Mueller, Konstanze Winklhofer, and Fulvio Reggiori

Corresponding author(s): Fulvio Reggiori (f.m.reggiori@aias.au.dk)

Review Timeline:

Submission Date:	5th Jan 26
Editorial Decision:	17th Feb 26
Revision Received:	8th Apr 26
Accepted:	24th Jun 26

Editor: Martina Rembold

Transaction Report:

(Note: With the exception of the correction of typographical or spelling errors that could be a source of ambiguity, letters and reports are not edited. Depending on transfer agreements, referee reports obtained elsewhere may or may not be included in this compilation. Referee reports are anonymous unless the Referee chooses to sign their reports.)

Dear Fulvio,

Thank you for the submission of your review article to EMBO reports. We have now received the full set of referee reports that are pasted below.

As you will see, the referees consider the review timely and of interest for the field, but they also have a number of suggestions how to strengthen it. Both referees comment on the limited information content of the figures and I fully agree with their assessment. Please create a new concept for the figures and redesign them to make full use of their potential to convey important concepts and biology.
In addition, please consider in particular the suggestions from referee #2 regarding the currently broad focus of your review article. The concern that the review reads a little bit like a summary of the data is important, and a more insightful discussion of the data, the highlighting of important conceptual discoveries and relevant biology will certainly improve the review.

I think this is a very interesting review and while I appreciate that incorporating the referees' suggestions will still require a bit of work, I am convinced that the article is worth it and will benefit from it.

As for timing, , would it be possible for you to submit the revised version by mid of April?

I look forward to seeing a revised version of your manuscript when it is ready. Please let me know if you have questions or comments regarding the revision and timing.

With best wishes,

Martina

Martina Rembold, PhD
Senior Editor
EMBO reports

****

Referee #1:

This review is a comprehensive overview of the roles of ubiquitin E3 ligases in selective autophagy. Most of the E3 ligases identified to date are well covered, making this a timely and valuable review.

Specific comments

1. The figures are quite similar to each other and, given the space they occupy, do not convey a large amount of information. A summary table listing the E3 ligases, their substrates, and the types of ubiquitin chains might be more informative and reader-friendly. For lysophagy, however, schematic illustrations may still be useful because the localization of substrates is important.

2. Page 10: SAR can induce autophagy not only through FIP200-dependent mechanisms but also through TBK1-dependent mechanisms (PMID: 37207627) and WIPI-dependent mechanisms (PMIDs: 37621214 and 40715440).

3. Page 17: In Refs. 172, 173, and 174, SIAH1 and SIAH2 are shown to mono-ubiquitinate α-synuclein and promote its aggregation. However, none of these studies conclude that degradation is enhanced. This point should be clarified to avoid misunderstanding.

4. Page 25, Fig. 4G: Ref. 239 shows that LUBAC is not required for M1-ubiquitination of T. gondii.

5. Page 29, Fig. 5D: Ref. 267 demonstrates that TRIM14 promotes "deubiquitination" of K48-linked ubiquitin chains on cGAS, thereby inhibiting its degradation. TRIM14 does not function as an E3 ligase that promotes degradation, making this figure potentially misleading.

6. While it may not be practical to include all E3 ligases, several important ones are missing. Examples include:
- Mitophagy: TRIMa has been reported to promote mitophagy via ubiquitination of TBK1 (PMID: 38814780). ARIH1/HHARI (PMID: 28930681) and HUWE1 (PMID: 30217973) have also been reported.
- Xenophagy: Ref. 217 reports that ARIH1 promotes xenophagy by K48-ubiquitination of S. Typhimurium.
- Proteaphagy: In yeast, three E3 ligases-San1, Rsp5, and Hul5-have been reported to be involved in proteasome autophagy

(PMID: 35294869).
- Lysophagy: CUL4A-DDB1-WDFY1 (PMID: 36103833) and FBXO3 (PMID: 39875384) have been reported as E3 complexes involved in lysophagy.

7. Page 18, fourth line from the bottom: "TOLLI" should be corrected to "TOLLIP".

8. Fig. 4C: "TRIM31" should be corrected to "TRIM32".

9. gp78 in Fig. 2 and AMFR in Fig. 5C refer to the same protein.

10. CHIP and STUB1 in Figs. 3A, 3B, 4E, and 5A also refer to the same protein.

11. TRIM11 is missing from Fig. 5D.

Referee #2:

The authors have written a detailed review of ubiquitin-dependent selective autophagy. The area is of great interest as cargo ubiquitylation is of utmost importance for cellular homeostasis, for the cellular defence against infections and for the ability to avoid neurodegenerative (and other) diseases. The area is under intense investigation but large gaps in our knowledge remain. The review is therefore timely.

The current manuscript is very long. In addition to detailed mechanistic discussions, the text also covers more peripheral topics in significant length. For example an extended discussion of chaperones. I would encourage the authors to critically evaluate the overall content of their review, and (most importantly!) to highlight important conceptual discoveries. The reader of this review will want to know what the experts consider relevant. A mere list of all findings that were ever made in this field is probably not in the readers' interest. (See for example page10 for the role of cargo receptors and compare with my suggestions in point9 below.)

Regarding figures, Fig1 provides only general knowledge and the information content of Figs2-5 is rather limited. To the extent that a plain table could provide similar information in a more condensed way. For the figures (whenever possible), it might be good to illustrate biology as an ordered process of events. For example how pink-parkin dependent mitophagy really works, or how S-guanylation and ubiquitylation of S.pyogenes are connected.

Specific points below:
1. Title: 'selective types of macroautophagy'. Taken literally the phrase suggest there are several types of macroautophagy, possibly even mechanistically distinct variants of macroautophagy. The authors should clarify what these 'selective types of macroautophagy are' and whey they decided to coin a term for their title that they then disband in the first line of the abstract ("... selective types of autophagy, hereafter selective autophagy").

2. Abstract: The authors suggest "The selectivity of these processes (i.e. macroautophagy) is guaranteed by selective autophagy receptors". This seems misleading. While there are truly selective cargo-receptors (for example NDP52 and TAX1BP1 targeting galectin-8 positive structures), in ub-dependent selective macroautophagy (the topic of this essay) cargo-selectivity is clearly established by E3 ligases, not by cargo receptors.

3. 'selective autophagy receptors' - a common term in this essay. The phrase suggests there might be other autophagy receptors, perhaps even 'non-selective autophagy receptors'? Perhaps the term cargo receptors would be better?

4. Line numbers would have been really helpful.

5. Page 3, bottom half: How do we know that the main function of ub is to serve as a tag for substrate recognition?

6. Page 3: the description of ubiquitination as a post-translational modification is too narrow given the recent discovery of a variety of non-proteinaceous substrates. I think this would be the kind of recent progress readers of this review might be interested in?

7. Page 5: In addition to HECT, RING and RBR ligases, other classes exist: IpaH, RZ

8. Page7, middle: 'Macroautophagy, hereafter autophagy' has been written thousands of times in the autophagy literature. To me, shortening macroautophagy to autophagy is the original sin of our field. The term beautifully describes processes that involve phagophores, thus effortlessly distinguishing macroautophagy from all forms of 'non-canonical autophagy' where ATG8 is conjugated to single membranes.

9. P10, bottom: The description of SAR function and phagophore formation is superficial and falls short of conceptualizing

established knowledge. I believe it is well established that SARs in selective autophagy choreograph multiple activities in a sequential manner: i) cargo binding, ii) recruitment of upstream ATGs (ULK complex, TBK1 complex) to initiate phagophore formation in situ, iii) directed growth of LC3 pos membrane around the cargo through LC3-LIR interactions.

10. P11: '...ub-independent SARs are part of the targeted structures, the ub-dependent SARs are soluble'. This simplification is not true - for example the recruitment of NDP52 and TAX1BP1 to Gal8 positive membranes.

11. Page15: is it necessary to discuss chaperones and the folding of proteins?

12. Page 20: It might not be advisable to assign intention to protein function. For example 'S.pyogenes ... secretes SLO... to infiltrate the cytoplasm'.

13. Page21 bottom: Ref220 does not report autoubiquitylation of RNF213 as a means to activate its anti-bacterial ligases activity.

14. Page22: Listeria is highlighted as producing cycli-di-AMP to antagonize STING, while other bacteria are suggested to have developed different strategies. It should be noted that many bacteria produce cyclic-di-AMP to regulate their own physiology, so Listeria is in no way special here.

Point-by-point rebuttal

We thank the two reviewers for their excellent and constructive criticisms, which have helped to improve and complete our manuscript. A point-by-point response to each reviewer's comment is given below. Major additions and changes in the manuscript are highlighted in blue.

Referee #1

1. The figures are quite similar to each other and, given the space they occupy, do not convey a large amount of information. A summary table listing the E3 ligases, their substrates, and the types of ubiquitin chains might be more informative and reader-friendly. For lysophagy, however, schematic illustrations may still be useful because the localization of substrates is important.

As requested, Figures 2 to 5 have been converted into Table 1, which lists the substrates, the E3 Ub ligases modifying them, the types of Ub chains appended and the related references. Schematic illustrations of lysophagy are now depicted in the new Figure 4.

2. Page 10: SAR can induce autophagy not only through FIP200-dependent mechanisms but also through TBK1-dependent mechanisms (PMID: 37207627) and WIPI-dependent mechanisms (PMIDs: 37621214 and 40715440).

Thank you for this remark. This information has now been mentioned on page 10, lines 250-255.

3. Page 17: In Refs. 172, 173, and 174, SIAH1 and SIAH2 are shown to mono-ubiquitinate α-synuclein and promote its aggregation. However, none of these studies conclude that degradation is enhanced. This point should be clarified to avoid misunderstanding.

This point has been clarified on page 17, lines 447-449.

4. Page 25, Fig. 4G: Ref. 239 shows that LUBAC is not required for M1-ubiquitination of T. gondii.

This has been corrected.

5. Page 29, Fig. 5D: Ref. 267 demonstrates that TRIM14 promotes "deubiquitination" of K48-linked ubiquitin chains on cGAS, thereby inhibiting its degradation. TRIM14 does not function as an E3 ligase that promotes degradation, making this figure potentially misleading.

Thank you for pointing this out. We have removed this information, which was indeed incorrect.

6. While it may not be practical to include all E3 ligases, several important ones are missing. Examples include:

- Mitophagy: TRIMa has been reported to promote mitophagy via ubiquitination of TBK1 (PMID: 38814780). ARIH1/HHARI (PMID: 28930681) and HUWE1 (PMID: 30217973) have also been reported.
- Xenophagy: Ref. 217 reports that ARIH1 promotes xenophagy by K48-ubiquitination of S. Typhimurium.
- Proteaphagy: In yeast, three E3 ligases-San1, Rsp5, and Hul5-have been reported to be involved in proteasome autophagy (PMID: 35294869).
- Lysophagy: CUL4A-DDB1-WDFY1 (PMID: 36103833) and FBXO3 (PMID: 39875384) have been reported as E3 complexes involved in lysophagy.

The roles of TRIM5a, ARIH1 and HUWE1 in mitophagy have been presented on page 15, lines 349-353, page 16, lines 371-375, and page 16, lines 375-380, respectively. The information of ARIH1 promoting xenophagy of S. Typhimurium has been added on page 23, lines 546-549. The trio of yeast E3 Ub ligases involed in proteaphagy has been described on page 28, lines 671-673. CUL4A-DDB1-WDFY1 and FBXO3 have now been described on page 30, lines 711-718.

7. Page 18, fourth line from the bottom: "TOLLI" should be corrected to "TOLLIP".

Corrected.

8. Fig. 4C: "TRIM31" should be corrected to "TRIM32".

Corrected.

9. gp78 in Fig. 2 and AMFR in Fig. 5C refer to the same protein.

GP78 has been changed into AMFR throughout the manuscript.

10. CHIP and STUB1 in Figs. 3A, 3B, 4E, and 5A also refer to the same protein.

STUB1 has been changed into CHIP throughout the manuscript.

11. TRIM11 is missing from Fig. 5D.

TRIM11 has been inserted in Table 1.

Referee #2

The current manuscript is very long. In addition to detailed mechanistic discussions, the text also covers more peripheral topics in significant length. For example an extended discussion of chaperones. I would encourage the authors to critically evaluate the overall content of their review, and (most importantly!) to highlight important conceptual discoveries. The reader of this review will want to know what the experts consider relevant. A mere list of all findings that were ever made in this field is probably not in the readers' interest. (See for example page10 for the role of cargo receptors and compare with my suggestions in point9 below.)

Thank you for this valuable feedback. The draft has been critically assessed, and several parts that were not relevant for the review per se have been removed. Additionally, concepts and better contextualizations have been added to highlight important points and eliminate mere lists.

Regarding figures, Fig1 provides only general knowledge and the information content of Figs2-5 is rather limited. To the extent that a plain table could provide similar information in a more condensed way. For the figures (whenever possible), it might be good to illustrate biology as an ordered process of events. For example how pink-parkin dependent mitophagy really works, or how S-guanylation and ubiquitylation of S. pyogenes are connected.

Figures 2 to 5 have been converted into Table 1, which lists the substrates, the E3 Ub ligases modifying them, the types of Ub chains appended and the related references. PINK1/PARKIN-dependent mitophagy, and the evolutionary competition in xenophagy are now depicted in the new Figures 2 and 3, respectively.

1. Title: 'selective types of macroautophagy'. Taken literally the phrase suggest there are several types of macroautophagy, possibly even mechanistically distinct variants of macroautophagy. The authors should clarify what these 'selective types of macroautophagy are' and whey they decided to coin a term for their title that they then disband in the first line of the abstract ("... selective types of autophagy, hereafter selective autophagy").

Thank you for the suggestion. The term has been defined in the abstract and the wording "hereafter autophagy" has been removed from the manuscript.

2. Abstract: The authors suggest "The selectivity of these processes (i.e. macroautophagy) is guaranteed by selective autophagy receptors". This seems misleading. While there are truly selective cargo-receptors (for example NDP52 and TAX1BP1 targeting galectin-8 positive structures), in ub-dependent selective macroautophagy (the topic of this essay) cargo-selectivity is clearly established by E3 ligases, not by cargo receptors.

Thank you for this comment. This point has been clarified in the abstract.

3. 'selective autophagy receptors' - a common term in this essay. The phrase suggests there might be other autophagy receptors, perhaps even 'non-selective autophagy receptors'? Perhaps the term cargo receptors would be better?

Thank you for this remark. The term SAR has been extensively used in the field of autophagy and refers to cargo receptors that during selective autophagy confer specificity for ubiquitylated substrates, rather than implying the existence of separate 'non-selective' autophagy receptors. In this review, we aligned with this nomenclature. Nonetheless, on page 10, line 215, we have now indicated that SARs can also referred to as cargo receptors.

4. Line numbers would have been really helpful.

Line numbers have been added.

5. Page 3, bottom half: How do we know that the main function of ub is to serve as a tag for substrate recognition?

We agree that this formulation is misleading. We have rephrased it on page 3, lines 52-53, and on page 4, lines 69-72.

6. Page 3: the description of ubiquitination as a post-translational modification is too narrow given the recent discovery of a variety of non-proteinaceous substrates. I think this would be the kind of recent progress readers of this review might be interested in?

Thank you for this suggestion. We have extened this paragraph on page 4, lines 69-72.

7. Page 5: In addition to HECT, RING and RBR ligases, other classes exist: IpaH, RZ

Thank you for pointing this out. These as well as two other recently discovered classes of E3 Ub ligases are now described on page 6, lines 124–132.

8. Page7, middle: 'Macroautophagy, hereafter autophagy' has been written thousands of times in the autophagy literature. To me, shortening macroautophagy to autophagy is the original sin of our field. The term beautifully describes processes that involve phagophores, thus effortlessly distinguishing macroautophagy from all forms of 'non-canonical autophagy' where ATG8 is conjugated to single membranes.

This sentence has been removed, and the prefix "macro" has been added where appropriate to distinguish macroautophagy from other types of autophagy.

9. P10, bottom: The description of SAR function and phagophore formation is superficial and falls short of conceptualizing established knowledge. I believe it is well established that SARs in selective autophagy choreograph multiple activities in a sequential manner: i) cargo binding, ii) recruitment of upstream ATGs (ULK complex, TBK1 complex) to initiate phagophore formation in situ, iii) directed growth of LC3 pos membrane around the cargo through LC3-LIR interactions.

Thank you for this comment. We have ameliorated the description of SARs' functions on page 11.

10. P11: '...ub-independent SARs are part of the targeted structures, the ub-dependent SARs are soluble'. This simplification is not true - for example the recruitment of NDP52 and TAX1BP1 to Gal8 positive membranes.

We agree. We have changed the sentence ¨While the Ub-independent SARs are part of the targeted structures, Ub-dependent SARs are soluble and recruited to specific cargo by binding

Ub chains via their Ub-binding domains (UBDs)¨ into ¨SARs bind cargo either directly, as in the case of organelle-resident SARs, or indirectly as the soluble SARs, which are recruited to specific cargoes by binding Ub chains via their Ub-binding domains (UBDs) or specific proteins such as GAL8.¨ on page 10, lines 239-242.

11. Page15: is it necessary to discuss chaperones and the folding of proteins?

This paragraph has been removed from the manuscript.

12. Page 20: It might not be advisable to assign intention to protein function. For example 'S.pyogenes ... secretes SLO... to infiltrate the cytoplasm'.

Thank you for this comment. This sentence has been corrected as well as others that had a similar construction.

13. Page21 bottom: Ref220 does not report autoubiquitylation of RNF213 as a means to activate its anti-bacterial ligases activity.

Thank you for this comment; autoubiquitination has been removed.

14. Page22: Listeria is highlighted as producing cycli-di-AMP to antagonize STING, while other bacteria are suggested to have developed different strategies. It should be noted that many bacteria produce cyclic-di-AMP to regulate their own physiology, so Listeria is in no way special here.

This point has been clarified on page 22, lines 584-585.

1st Revision - Editorial Decision **24th Jun 2026**

Prof. Fulvio Reggiori
Aarhus University
Department of Bomedicne
Aarhus
Denmark

Dear Fulvio,

Thank you for implementing the suggested editorial edits and for sending the final manuscript files and figures. We have uploaded these for you and I am now pleased to accept your review article for publication in EMBO reports. I have sent your figures to the team of graphics editors we work with and they should contact you within the next 10 working days with the drafts. Once the figures have been approved and are ready for publication, we will export your article to our production team.

Your manuscript will then be processed for publication by EMBO Press. It will be copy edited and you will receive page proofs prior to publication. Please note that you will be contacted by Springer Nature Author Services to complete licensing information.

Should you be planning a Press Release on your article, please notify the production team via this form in order to coordinate publication and release dates: https://forms.cloud.microsoft/e/eH4A8JVP6g

If you have any questions, please do not hesitate to contact the Editorial Office. Thank you for your contribution to EMBO Reports. It was a pleasure working with you on this article.

Best regards,

Martina

Martina Rembold, PhD
Senior Editor
EMBO reports

Referee #2:

The authors have made every effort to address my comments.

One final comment - in Fig3A LPS seems to be displayed on the surface of Streptococcus.

>>> Please note that it is EMBO Reports policy for the transcript of the editorial process (containing referee reports and your response letter) to be published as an online supplement to each paper. If you do NOT want this, you will need to inform the Editorial Office via email immediately. More information is available here: https://link.springer.com/partners/embo-press/editorial-policies#Peer%20review
